# 2-(4-Chloro­phen­yl)-2-oxoethyl 2-meth­oxy­benzoate

**DOI:** 10.1107/S1600536811021246

**Published:** 2011-06-18

**Authors:** Hoong-Kun Fun, Safra Izuani Jama Asik, B. Garudachari, Arun M. Isloor, M. N. Satyanarayan

**Affiliations:** a X-ray Crystallography Unit, School of Physics, Universiti Sains Malaysia, 11800 USM, Penang, Malaysia; b Organic Electronics Division, Department of Chemistry, National Institute of Technology–Karnataka, Surathkal, Mangalore 575 025, India; c Department of Physics, National Institute of Technology–Karnataka, Surathkal, Mangalore 575 025, India

## Abstract

In the title compound, C_16_H_13_ClO_4_, the two benzene rings make a dihedral angle of 86.38 (8)°. In the crystal, inter­molecular C—H⋯O hydrogen bonds link the mol­ecules to form columns along the *a* axis. The mol­ecules are also stabilized by a π–π stacking inter­action, with a centroid–centroid distance of 3.7793 (10) Å between the inversion-related benzene rings.

## Related literature

For general background to phenacyl benzoates, see: Rather & Reid (1919 ▶); Sheehan & Umezaw (1973 ▶); Ruzicka *et al.* (2002 ▶); Litera *et al.* (2006 ▶). For applications and synthesis of oxazoles, imidazoles and benzoxazepines, see: Huang *et al.* (1996 ▶); Gandhi *et al.* (1995 ▶). For bond-length data, see: Allen *et al.* (1987 ▶).
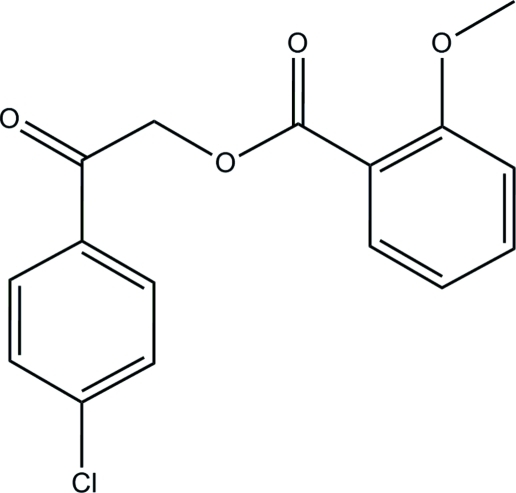


## Experimental

### 

#### Crystal data

C_16_H_13_ClO_4_
*M* *_r_* = 304.71
Orthorhombic, 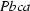

*a* = 7.7207 (6) Å
*b* = 14.4411 (12) Å
*c* = 26.064 (2) Å
*V* = 2906.0 (4) Å^3^
*Z* = 8
Mo *K*α radiation
μ = 0.28 mm^−1^
*T* = 296 K
0.51 × 0.29 × 0.19 mm

#### Data collection

Bruker APEXII DUO CCD area-detector diffractometer
Absorption correction: multi-scan (*SADABS*; Bruker, 2009 ▶) *T*_min_ = 0.829, *T*_max_ = 0.950
16215 measured reflections
4027 independent reflections
2731 reflections with *I* > 2σ(*I*)
*R*_int_ = 0.025

#### Refinement

*R*[*F* ^2^ > 2σ(*F* ^2^)] = 0.041
*wR*(*F* ^2^) = 0.116
*S* = 1.02
4027 reflections
190 parameters
H-atom parameters constrained
Δρ_max_ = 0.17 e Å^−3^
Δρ_min_ = −0.35 e Å^−3^

### 

Data collection: *APEX2* (Bruker, 2009 ▶); cell refinement: *SAINT* (Bruker, 2009 ▶); data reduction: *SAINT*; program(s) used to solve structure: *SHELXTL* (Sheldrick, 2008 ▶); program(s) used to refine structure: *SHELXTL*; molecular graphics: *SHELXTL*; software used to prepare material for publication: *SHELXTL* and *PLATON* (Spek, 2009 ▶).

## Supplementary Material

Crystal structure: contains datablock(s) global, I. DOI: 10.1107/S1600536811021246/is2727sup1.cif

Structure factors: contains datablock(s) I. DOI: 10.1107/S1600536811021246/is2727Isup2.hkl

Supplementary material file. DOI: 10.1107/S1600536811021246/is2727Isup3.cml

Additional supplementary materials:  crystallographic information; 3D view; checkCIF report

## Figures and Tables

**Table 1:** Hydrogen-bond geometry (Å, °)

*D*—H⋯*A*	*D*—H	H⋯*A*	*D*⋯*A*	*D*—H⋯*A*
C2—H2*A*⋯O1^i^	0.93	2.40	3.301 (2)	164

Symmetry code: (i) 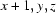.

**Fig. 1.**
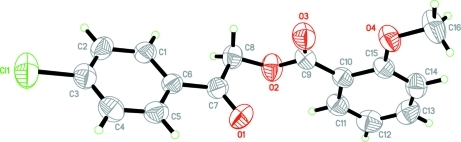
The structure of the title compound, showing 50% probability displacement ellipsoids and the atom-numbering scheme.

**Fig. 2.**
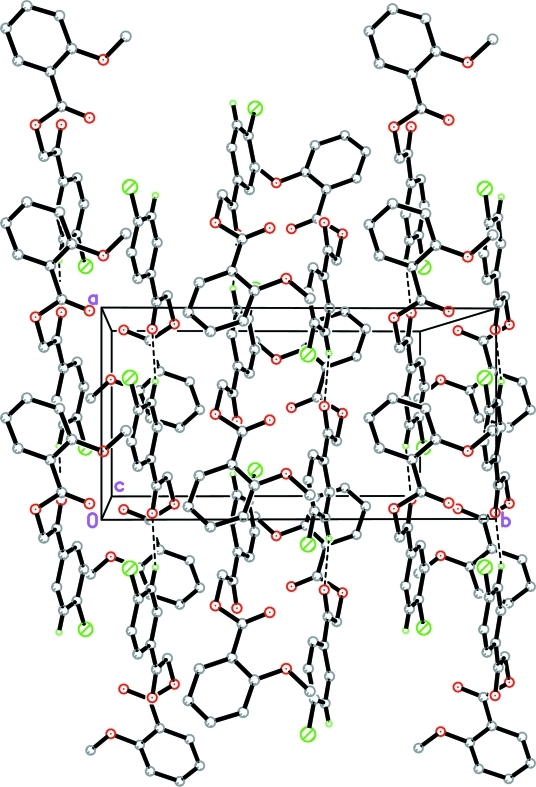
The crystal packing, viewed along the c axis, showing columns down to the a axis. Hydrogen atoms that are not involved in hydrogen bonding (dashed lines) are omitted for clarity.

## supplementary crystallographic information

### Comment

Phenacyl benzoate derivatives are very important in identification of organic acids (Rather & Reid, 1919) as they undergo photolysis in neutral and mild conditions (Sheehan & Umezaw, 1973; Ruzicka *et al.*, 2002 ; Litera *et al.*, 2006). They find applications in the field of synthetic chemistry for the synthesis of oxazoles, imidazoles (Huang *et al.*, 1996) and benzoxazepines (Gandhi *et al.*, 1995). The phenacyl esters are usually prepared by reaction between acids with phenacylbromide derivatives in DMF using sodium or potassium carbonate as base. We hereby report the crystal structure of 2-(4-chlorophenyl)-2-oxoethyl 2-methoxybenzoate, (I), which has potential commercial importance.

In the title compound of (I), the bond lengths (Allen *et al.*, 1987) and angles show the normal values. The two benzene rings (C1–C6 and C10–C15) make a dihedral angle of 86.38 (8)°.

In the crystal packing (Fig. 2), intermolecular C2—H2A···O1 hydrogen bonds (Table 1) link the molecules to form columns down to the *a*-axis. The molecules are also stabilized by π–π stacking interactions between the inversion-related benzene rings (C1–C6 ; centroid *Cg*1) with a *Cg*1···*Cg*1^ii^ separation of 3.7793 (10) Å [symmetry code: (ii) 2 - *x*, -*y*, 2 - *z*].

### Experimental

The mixture of 2-methoxybenzoic acid (1.0 g, 0.0065 mol), potassium carbonate (0.98 g, 0.0071 mol) and 2-bromo-1-(4-chlorophenyl)ethanone (1.45 g, 0.0065 mol) in dimethylformamide (10 ml) was stirred at room temperature for 2 h. On cooling, colorless needle-shaped crystals of 2-(4-chlorophenyl)-2-oxoethyl 2-methoxybenzoate begins to separate out. It was collected by filtration and recrystallized from ethanol. Yield : 1.9 g, 95 %, *M.p.* : 391–392 K, (CAS Registry Number: 282714–31–2).

### Refinement

All H atoms were placed in calculated positions with C–H = 0.93–0.97 Å. The *U*_iso_(H) values were constrained to be 1.5*U*_eq_ of the carrier atom for methyl H atoms and 1.2*U*_eq_ for the remaining H atoms.

### Crystal data

**Table:**

C_16_H_13_ClO_4_	*F*(000) = 1264
*M**_r_* = 304.71	*D*_x_ = 1.393 Mg m^−^^3^
Orthorhombic, *P**b**c**a*	Mo *K*α radiation, λ = 0.71073 Å
Hall symbol: -P 2ac 2ab	Cell parameters from 3855 reflections
*a* = 7.7207 (6) Å	θ = 2.9–29.2°
*b* = 14.4411 (12) Å	µ = 0.28 mm^−^^1^
*c* = 26.064 (2) Å	*T* = 296 K
*V* = 2906.0 (4) Å^3^	Block, colourless
*Z* = 8	0.51 × 0.29 × 0.19 mm

### Data collection

**Table:**

Bruker APEXII DUO CCD area-detector diffractometer	4027 independent reflections
Radiation source: fine-focus sealed tube	2731 reflections with *I* > 2σ(*I*)
graphite	*R*_int_ = 0.025
φ and ω scans	θ_max_ = 29.5°, θ_min_ = 2.9°
Absorption correction: multi-scan (*SADABS*; Bruker, 2009)	*h* = −10→9
*T*_min_ = 0.829, *T*_max_ = 0.950	*k* = −14→20
16215 measured reflections	*l* = −36→35

### Refinement

**Table:**

Refinement on *F*^2^	Primary atom site location: structure-invariant direct methods
Least-squares matrix: full	Secondary atom site location: difference Fourier map
*R*[*F*^2^ > 2σ(*F*^2^)] = 0.041	Hydrogen site location: inferred from neighbouring sites
*wR*(*F*^2^) = 0.116	H-atom parameters constrained
*S* = 1.02	*w* = 1/[σ^2^(*F*_o_^2^) + (0.0471*P*)^2^ + 0.6504*P*] where *P* = (*F*_o_^2^ + 2*F*_c_^2^)/3
4027 reflections	(Δ/σ)_max_ = 0.001
190 parameters	Δρ_max_ = 0.17 e Å^−^^3^
0 restraints	Δρ_min_ = −0.35 e Å^−^^3^

### Special details

**Table:**

Geometry. All esds (except the esd in the dihedral angle between two l.s. planes) are estimated using the full covariance matrix. The cell esds are taken into account individually in the estimation of esds in distances, angles and torsion angles; correlations between esds in cell parameters are only used when they are defined by crystal symmetry. An approximate (isotropic) treatment of cell esds is used for estimating esds involving l.s. planes.
Refinement. Refinement of F^2^ against ALL reflections. The weighted R-factor wR and goodness of fit S are based on F^2^, conventional R-factors R are based on F, with F set to zero for negative F^2^. The threshold expression of F^2^ > 2sigma(F^2^) is used only for calculating R-factors(gt) etc. and is not relevant to the choice of reflections for refinement. R-factors based on F^2^ are statistically about twice as large as those based on F, and R- factors based on ALL data will be even larger.

### Fractional atomic coordinates and isotropic or equivalent isotropic displacement parameters (Å^2^)

**Table:**

	*x*	*y*	*z*	*U*_iso_*/*U*_eq_	
Cl1	1.18791 (7)	0.06652 (3)	1.10182 (2)	0.08142 (19)	
O1	0.45975 (15)	0.12921 (11)	0.96747 (5)	0.0746 (4)	
O2	0.49777 (16)	0.19520 (7)	0.87365 (5)	0.0624 (3)	
O3	0.46624 (16)	0.04491 (8)	0.85569 (5)	0.0667 (3)	
O4	0.19233 (15)	0.00492 (7)	0.79740 (4)	0.0600 (3)	
C1	0.9263 (2)	0.14336 (11)	0.97782 (6)	0.0523 (4)	
H1A	0.9515	0.1678	0.9457	0.063*	
C2	1.0596 (2)	0.12582 (11)	1.01201 (7)	0.0571 (4)	
H2A	1.1739	0.1382	1.0031	0.068*	
C3	1.0198 (2)	0.08973 (10)	1.05937 (6)	0.0546 (4)	
C4	0.8517 (2)	0.07047 (11)	1.07348 (7)	0.0582 (4)	
H4A	0.8276	0.0459	1.1057	0.070*	
C5	0.7198 (2)	0.08809 (11)	1.03928 (6)	0.0534 (4)	
H5A	0.6059	0.0753	1.0485	0.064*	
C6	0.75479 (19)	0.12480 (10)	0.99111 (6)	0.0455 (3)	
C7	0.6086 (2)	0.14152 (10)	0.95508 (6)	0.0504 (4)	
C8	0.6513 (2)	0.17422 (11)	0.90174 (6)	0.0543 (4)	
H8A	0.7236	0.2290	0.9037	0.065*	
H8B	0.7159	0.1265	0.8839	0.065*	
C9	0.4084 (2)	0.12187 (10)	0.85521 (5)	0.0487 (3)	
C10	0.2379 (2)	0.15154 (10)	0.83489 (5)	0.0462 (3)	
C11	0.1787 (2)	0.24124 (11)	0.84420 (6)	0.0566 (4)	
H11A	0.2469	0.2813	0.8635	0.068*	
C12	0.0216 (3)	0.27210 (13)	0.82551 (7)	0.0672 (5)	
H12A	−0.0156	0.3322	0.8321	0.081*	
C13	−0.0790 (2)	0.21275 (13)	0.79706 (7)	0.0669 (5)	
H13A	−0.1847	0.2331	0.7842	0.080*	
C14	−0.0255 (2)	0.12352 (12)	0.78741 (6)	0.0594 (4)	
H14A	−0.0954	0.0842	0.7682	0.071*	
C15	0.1320 (2)	0.09184 (10)	0.80609 (5)	0.0475 (3)	
C16	0.0868 (3)	−0.05690 (13)	0.76827 (8)	0.0822 (6)	
H16A	0.1445	−0.1155	0.7650	0.123*	
H16B	0.0669	−0.0313	0.7348	0.123*	
H16C	−0.0221	−0.0655	0.7854	0.123*	

### Atomic displacement parameters (Å^2^)

**Table:**

	*U*^11^	*U*^22^	*U*^33^	*U*^12^	*U*^13^	*U*^23^
Cl1	0.0864 (4)	0.0640 (3)	0.0939 (4)	0.0087 (2)	−0.0384 (3)	−0.0086 (2)
O1	0.0402 (6)	0.1070 (11)	0.0766 (8)	−0.0079 (7)	0.0051 (6)	−0.0064 (7)
O2	0.0648 (7)	0.0453 (6)	0.0772 (8)	−0.0033 (5)	−0.0195 (6)	−0.0001 (5)
O3	0.0694 (8)	0.0505 (6)	0.0802 (8)	0.0105 (6)	−0.0215 (6)	−0.0133 (5)
O4	0.0707 (8)	0.0479 (6)	0.0615 (6)	0.0005 (5)	−0.0169 (5)	−0.0098 (5)
C1	0.0459 (8)	0.0574 (8)	0.0537 (8)	−0.0062 (7)	0.0056 (7)	−0.0057 (7)
C2	0.0407 (8)	0.0584 (9)	0.0721 (10)	−0.0042 (7)	0.0000 (7)	−0.0136 (8)
C3	0.0601 (10)	0.0413 (7)	0.0623 (9)	0.0040 (7)	−0.0108 (8)	−0.0124 (6)
C4	0.0712 (11)	0.0506 (8)	0.0529 (9)	−0.0036 (8)	0.0003 (8)	−0.0043 (7)
C5	0.0501 (9)	0.0535 (8)	0.0566 (9)	−0.0060 (7)	0.0095 (7)	−0.0071 (7)
C6	0.0412 (7)	0.0440 (7)	0.0513 (8)	−0.0034 (6)	0.0038 (6)	−0.0112 (6)
C7	0.0443 (8)	0.0480 (7)	0.0590 (9)	−0.0049 (7)	0.0024 (7)	−0.0121 (6)
C8	0.0497 (9)	0.0506 (8)	0.0626 (9)	−0.0059 (7)	−0.0071 (7)	−0.0005 (7)
C9	0.0575 (9)	0.0461 (8)	0.0426 (7)	−0.0003 (7)	−0.0033 (6)	−0.0024 (6)
C10	0.0542 (8)	0.0453 (7)	0.0390 (7)	0.0017 (6)	−0.0006 (6)	0.0012 (6)
C11	0.0667 (10)	0.0495 (8)	0.0535 (8)	0.0043 (8)	−0.0017 (7)	−0.0030 (7)
C12	0.0761 (12)	0.0580 (10)	0.0674 (10)	0.0192 (9)	0.0019 (9)	0.0036 (8)
C13	0.0600 (10)	0.0755 (11)	0.0653 (10)	0.0117 (9)	−0.0070 (9)	0.0138 (9)
C14	0.0608 (10)	0.0640 (10)	0.0532 (9)	−0.0026 (8)	−0.0110 (7)	0.0066 (7)
C15	0.0560 (9)	0.0487 (7)	0.0377 (7)	0.0005 (7)	−0.0012 (6)	0.0033 (6)
C16	0.1015 (16)	0.0592 (10)	0.0859 (13)	−0.0046 (11)	−0.0344 (12)	−0.0164 (9)

### Geometric parameters (Å, °)

**Table:**

Cl1—C3	1.7380 (17)	C7—C8	1.505 (2)
O1—C7	1.2069 (19)	C8—H8A	0.9700
O2—C9	1.3521 (18)	C8—H8B	0.9700
O2—C8	1.426 (2)	C9—C10	1.482 (2)
O3—C9	1.1978 (18)	C10—C11	1.395 (2)
O4—C15	1.3579 (18)	C10—C15	1.406 (2)
O4—C16	1.428 (2)	C11—C12	1.381 (2)
C1—C2	1.384 (2)	C11—H11A	0.9300
C1—C6	1.395 (2)	C12—C13	1.374 (3)
C1—H1A	0.9300	C12—H12A	0.9300
C2—C3	1.375 (2)	C13—C14	1.376 (3)
C2—H2A	0.9300	C13—H13A	0.9300
C3—C4	1.377 (3)	C14—C15	1.388 (2)
C4—C5	1.377 (2)	C14—H14A	0.9300
C4—H4A	0.9300	C16—H16A	0.9600
C5—C6	1.390 (2)	C16—H16B	0.9600
C5—H5A	0.9300	C16—H16C	0.9600
C6—C7	1.488 (2)		
			
C9—O2—C8	116.12 (12)	H8A—C8—H8B	108.0
C15—O4—C16	118.10 (13)	O3—C9—O2	122.20 (15)
C2—C1—C6	120.70 (15)	O3—C9—C10	127.08 (14)
C2—C1—H1A	119.6	O2—C9—C10	110.71 (12)
C6—C1—H1A	119.6	C11—C10—C15	118.16 (14)
C3—C2—C1	118.78 (15)	C11—C10—C9	119.82 (14)
C3—C2—H2A	120.6	C15—C10—C9	122.02 (13)
C1—C2—H2A	120.6	C12—C11—C10	121.74 (16)
C2—C3—C4	121.79 (15)	C12—C11—H11A	119.1
C2—C3—Cl1	118.55 (14)	C10—C11—H11A	119.1
C4—C3—Cl1	119.66 (14)	C13—C12—C11	119.05 (16)
C3—C4—C5	119.14 (16)	C13—C12—H12A	120.5
C3—C4—H4A	120.4	C11—C12—H12A	120.5
C5—C4—H4A	120.4	C12—C13—C14	120.86 (17)
C4—C5—C6	120.75 (15)	C12—C13—H13A	119.6
C4—C5—H5A	119.6	C14—C13—H13A	119.6
C6—C5—H5A	119.6	C13—C14—C15	120.51 (17)
C5—C6—C1	118.84 (15)	C13—C14—H14A	119.7
C5—C6—C7	118.98 (14)	C15—C14—H14A	119.7
C1—C6—C7	122.17 (14)	O4—C15—C14	123.15 (14)
O1—C7—C6	121.98 (15)	O4—C15—C10	117.17 (14)
O1—C7—C8	120.12 (15)	C14—C15—C10	119.67 (15)
C6—C7—C8	117.90 (13)	O4—C16—H16A	109.5
O2—C8—C7	111.06 (14)	O4—C16—H16B	109.5
O2—C8—H8A	109.4	H16A—C16—H16B	109.5
C7—C8—H8A	109.4	O4—C16—H16C	109.5
O2—C8—H8B	109.4	H16A—C16—H16C	109.5
C7—C8—H8B	109.4	H16B—C16—H16C	109.5
			
C6—C1—C2—C3	0.0 (2)	C8—O2—C9—C10	170.17 (13)
C1—C2—C3—C4	0.2 (2)	O3—C9—C10—C11	170.24 (16)
C1—C2—C3—Cl1	179.21 (12)	O2—C9—C10—C11	−10.50 (19)
C2—C3—C4—C5	−0.2 (2)	O3—C9—C10—C15	−9.9 (2)
Cl1—C3—C4—C5	−179.21 (12)	O2—C9—C10—C15	169.40 (13)
C3—C4—C5—C6	0.0 (2)	C15—C10—C11—C12	−0.7 (2)
C4—C5—C6—C1	0.2 (2)	C9—C10—C11—C12	179.24 (15)
C4—C5—C6—C7	179.14 (14)	C10—C11—C12—C13	0.1 (3)
C2—C1—C6—C5	−0.2 (2)	C11—C12—C13—C14	0.4 (3)
C2—C1—C6—C7	−179.12 (14)	C12—C13—C14—C15	−0.3 (3)
C5—C6—C7—O1	4.4 (2)	C16—O4—C15—C14	−1.0 (2)
C1—C6—C7—O1	−176.70 (15)	C16—O4—C15—C10	179.95 (16)
C5—C6—C7—C8	−175.29 (13)	C13—C14—C15—O4	−179.34 (15)
C1—C6—C7—C8	3.6 (2)	C13—C14—C15—C10	−0.3 (2)
C9—O2—C8—C7	−77.10 (17)	C11—C10—C15—O4	179.86 (13)
O1—C7—C8—O2	5.8 (2)	C9—C10—C15—O4	0.0 (2)
C6—C7—C8—O2	−174.59 (12)	C11—C10—C15—C14	0.7 (2)
C8—O2—C9—O3	−10.5 (2)	C9—C10—C15—C14	−179.15 (14)

### Hydrogen-bond geometry (Å, °)

**Table:**

*D*—H···*A*	*D*—H	H···*A*	*D*···*A*	*D*—H···*A*
C2—H2A···O1^i^	0.93	2.40	3.301 (2)	164.

Symmetry codes: (i) *x*+1, *y*, *z*.
